# Digital Health Information Use, Trust, and Language-Related Disparities in Health Literacy in South Tyrol, Italy: Cross-Sectional Survey Study

**DOI:** 10.2196/80934

**Published:** 2026-09-24

**Authors:** Dietmar Ausserhofer, Verena Barbieri, Stefano Lombardo, Timon Gärtner, Klaus Eisendle, Giuliano Piccoliori, Adolf Engl, Christian Josef Wiedermann

**Affiliations:** 1 Institute of General Practice and Public Health, Claudiana—College of Health Professions, Lorenz Böhler Street 13, Bolzano-Bozen, Bolzano, 39100, Italy, 39 0471 067 ext 392; 2 Provincial Institute for Statistics of the Autonomous Province of Bolzano - South Tyrol, Bolzano-Bozen, Bolzano, Italy; 3 Directorate, Claudiana—College of Health Professions, Bolzano-Bozen, Italy

**Keywords:** health literacy, digital health, multilingual, bilingual, language, health information, mediation, 16-item Health Literacy Survey European Union Questionnaire, HLS-EU-Q16, digital engagement, disparities

## Abstract

**Background:**

Health literacy (HL) is a crucial determinant of population health; however, disparities persist in multilingual settings. In South Tyrol, Italy, where German- and Italian-speaking populations coexist within a unified health care system, HL disparities may reflect differences in language, culture, and digital engagement.

**Objective:**

This study investigated whether the usage of digital health information mediates the relationship between language group and HL and examined informational trust and other sociodemographic factors as covariates of HL.

**Methods:**

Data were obtained from a population-based survey conducted in South Tyrol, Italy. The analytical sample comprised 1500 participants with adequate responses to the 16-item Health Literacy Survey European Union Questionnaire (HLS-EU-Q16) instrument and an identified language group. Mediation analysis was performed using PROCESS Model 4 (5000 bootstrap samples), with use of digital health information as the mediator, language group as the predictor, and HL as the outcome. Covariates included age, sex, education, self-rated health, trust in professional information sources, and trust in health information on the internet.

**Results:**

Italian-speaking participants showed significantly higher HL scores (mean 12.54, SD 3.31 vs mean 11.56, SD 3.48 on a 0-16 scale; *P*<.001) and reported more frequent digital health information use (*P*=.003) compared with German-speaking participants. Digital health information use was weakly positively correlated with HL in bivariate analyses (*r*=0.11). In the fully adjusted mediation model, the indirect effect of language group on HL through digital health information use was not significant (*B*=0.018, 95% bootstrap CI −0.035 to 0.074; partially standardized effect=0.005), whereas the direct effect of language group remained robust (*B*=0.96; *P*<.001). Trust in professional health information (*B*=0.39; *P*<.001) and trust in health information on the internet (*B*=0.61; *P*<.001) were both positively associated with HL.

**Conclusions:**

Although digital engagement and online trust are related to HL, they do not explain the persistent language-based disparities in HL observed in South Tyrol. Public health strategies should prioritize strengthening critical appraisal skills, fostering trust in professional health information sources, and improving digital access to effectively address HL inequalities in multilingual populations.

## Introduction

### Background

Health literacy (HL) is a crucial determinant of individual and public health, significantly affecting the capacity to access, comprehend, and use health-related information [1-3]. Low HL correlates with adverse health outcomes, diminished use of preventive services, and elevated health care costs [1]. In increasingly complex information environments, individuals must not only process health information but also assess its relevance and reliability. The advent of digital media has transformed public engagement with health information through the widespread availability of online resources and tools [4-6]. Repeated engagement with online health resources may foster HL by familiarizing users with medical terminology; supporting navigation of the health system; and providing recurring opportunities to locate, appraise, and apply health information, particularly when trustworthy sources are used [6-8]. At the same time, disparities in HL persist across Europe, often mirroring gradients of age, education, and social status [9,10]. In multilingual regions, such disparities may be further influenced by language-related differences in access to and trust in the information environment [11]. South Tyrol, a predominantly bilingual province in northern Italy where German and Italian are co-official languages, provides a particularly pertinent context for examining these dynamics: its shared health care infrastructure and coexisting language communities allow HL variations to be investigated under stable institutional and policy conditions.

### Prior Work

A recent study conducted by Ausserhofer et al [12] represents the inaugural population-based HL assessment in South Tyrol, using the 16-item Health Literacy Survey European Union Questionnaire (HLS-EU-Q16) instrument. The study identified significant disparities between the language groups, with Italian speakers exhibiting higher HL than German speakers. These differences persisted even after controlling for variables such as age, education, chronic illness, and professional background in health and social care. Notably, domain-specific analyses indicated that German speakers scored low in terms of health promotion (HP) literacy. Concurrently, Wiedermann et al [13] investigated the usage of health information and trust in the same regional context. Their findings suggest that Italian speakers use and trust digital health information sources more frequently, whereas German speakers predominantly rely on traditional or interpersonal sources. These variations in engagement with digital health information may elucidate HL disparities that are not solely attributable to sociodemographic factors. Collectively, these studies propose that South Tyrol serves as a natural model for examining the interaction between digital information behavior and linguistic background in shaping HL. The region’s bilingual infrastructure, shared health care system, and controlled regional variation provide a quasi-experimental setting for exploring the mechanisms underlying HL inequalities.

### Goal of This Study

This study investigated whether variations in engagement with digital health information mediate the relationship between language group (Italian vs German) and HL. Furthermore, we examined trust in health information from professional sources and internet-based platforms as potential independent correlates of HL. By examining behavior and perceptual factors associated with HL disparities, this study contributes to the formulation of targeted, culturally, and linguistically responsive public health strategies.

## Methods

### Study Design and Participants

This study used a cross-sectional survey conducted between March and May 2024 among residents of South Tyrol, a multilingual and autonomous province in northern Italy. Eligible participants were individuals aged 18 years or older, proficient in either German or Italian, and randomly selected from the provincial resident registry through stratified sampling based on language groups. The sample size was determined by the established survey methodology and operational expertise of the Provincial Institute of Statistics of the Autonomous Province of Bolzano-South Tyrol (ASTAT). A stratified random sample of 4000 adult residents of South Tyrol was drawn using the SURVEYSELECT procedure in SAS (version 9.2; SAS Institute Inc), with subsequent poststratification weighting performed in ReGenesees (version 2.3; Italian National Institute of Statistics). The stratification criteria included age group (18‐34, 35‐54, and 55+ years), sex, citizenship, and municipality of residence. The questionnaire was administered in a mixed mode design: participants received a bilingual postal invitation containing an individual access code to complete the questionnaire online via the LimeSurvey platform (LimeSurvey GmbH), and respondents who preferred to could instead complete an enclosed paper version and return it by post.

### Measures

Sociodemographic characteristics included self-reported sex (male or female), age (in years), and age group (18‐34, 35‐54, and 55‐99 years). Residence was classified as urban or rural based on municipal criteria. Educational attainment was categorized into 4 levels: middle school or less, vocational school, high school, and university. Participants also reported whether they were employed in the health or social care sector (yes or no).

Health status was evaluated using a single self-rated health item on a scale from 0 (worst imaginable health) to 100 (best imaginable health). The burden of chronic illness was assessed using a checklist of prevalent conditions, including cardiovascular, respiratory, metabolic, oncological, and mental health disorders. A binary indicator for the presence of any chronic condition was derived (yes or no), along with a variable indicating the number of chronic diseases (range: 0‐9) per respondent. These indicators have been previously documented [13].

Trust in professional health information was measured using a 4-item index reflecting trust in doctors, pharmacists, health institutions, and the information provided by them. The items were rated on a 4-point scale, ranging from “very much (1)” to “not at all (4),” coded inversely, and aggregated to form a trust index [13], with higher scores indicating greater trust. Trust in the internet was assessed separately with a single mistrust item, again ranging from “very much (1)” to “not at all (4),” and it was coded inversely as well.

Digital health information use was operationalized through 4 items assessing the frequency of using search engines, passive browsing, forums, and social media for health-related purposes, on a 4-item scale ranging from 1 (regularly) to 4 (never); item scores were inversely rescaled and summed to create a composite score (range: 4‐16), with higher scores indicating more frequent digital health information use [13].

HL was assessed using the validated HLS-EU-Q16 questionnaire, implemented in German and Italian, with validated translations that demonstrated robust psychometric properties [9,14]. All 16 items were scored on a 4-point difficulty scale, dichotomized according to standard procedures, and summed to yield a raw score of 0 to 16. Respondents with fewer than 14 valid responses were excluded from the HL-score calculation.

Consistent with the procedure reported by Ausserhofer et al [12], domain-specific scores were computed for the 3 HL dimensions represented in the HLS-EU-Q16: (1) health care (HC), (2) disease prevention (DP), and (3) HP. Each domain score was calculated by summing the dichotomized responses for the items assigned to the respective domains, as outlined in the HLS-EU conceptual framework. Only respondents with complete data (ie, all 16 items answered) were included in the domain-level analysis to ensure comparability across dimensions.

### Statistical Analysis

#### General Analytic Approach

All analyses were performed using IBM SPSS Statistics (version 29.0.2.0). Descriptive statistics were calculated for all variables stratified by language group. Because formal normality tests are overly sensitive at large sample sizes, the distributions of continuous variables were assessed using skewness and kurtosis together with visual inspection of histograms and Q-Q plots; all continuous measures entered into the analyses showed skewness within conventionally acceptable limits (|skewness|<2). Given the large analytic sample (N=1500) and the robustness of parametric procedures under these conditions, group differences in continuous variables were evaluated using independent-sample 2-tailed *t* tests with Welch correction, and bivariate associations were quantified using Pearson correlation coefficients. Group differences in categorical variables were evaluated using chi-square tests. Effect sizes were reported as Cramér *V* for categorical comparisons and Cohen *d* for continuous comparisons, with 2-tailed *P*<.05 considered statistically significant.

All estimates and tests were computed on the unweighted analytic sample, so that the descriptive, correlation, and mediation analyses would share a common basis; this was required because the PROCESS macro does not accommodate case weights. The poststratification weights (ReGenesees; Italian National Institute of Statistics) were retained only to document the representativeness of the realized sample (see “Study Design and Participants” section); applying them did not change the direction or statistical significance of any of the reported between-group differences in sociodemographic and health characteristics.

#### Covariate Selection

Covariates for the mediation model were selected a priori on theoretical and empirical grounds rather than through data-driven stepwise procedures. Age, sex, educational attainment, self-rated health status, trust in professional health information, and trust in health information on the internet were included as covariates in both the mediator and outcome equations, as each is an established correlate of HL as well as digital health information use [7-9,15-18]. Multicollinearity was acceptable in all models (variance inflation factor<1.2). Educational attainment, dichotomized for the analysis (high school or higher vs lower), was retained as a covariate despite its modest bivariate correlation, given its established theoretical relevance to both HL and digital engagement.

#### Mediation Analysis

**Figure 1.**
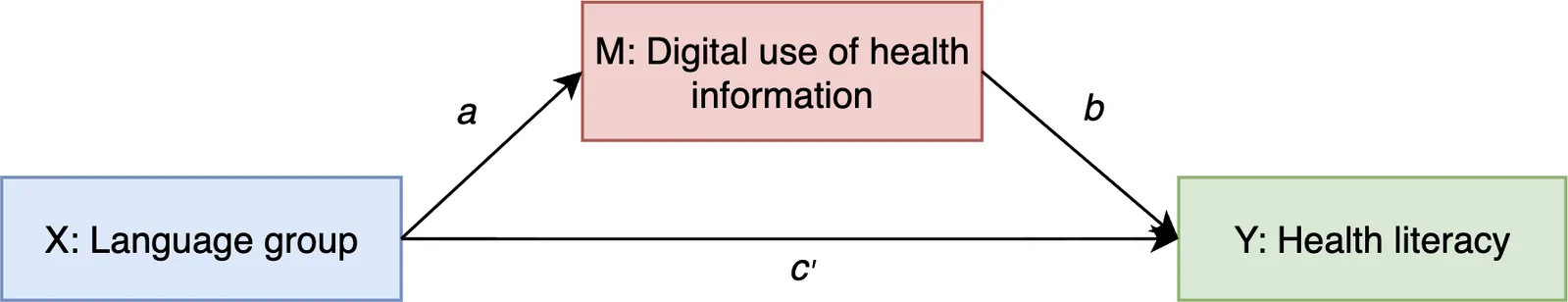
Conceptual model of the mediation analysis (PROCESS Model 4). The language group (X: German vs Italian) is modeled as a predictor of health literacy (Y) directly (path *c′*) and indirectly via the digital use of health information (M; paths *a* and *b*). The model tests whether differences in digital engagement account for language group disparities in health literacy.

We conducted a mediation analysis using Hayes’ PROCESS macro for SPSS (version 4.2; Model 4) [19] to examine whether the usage of digital health information mediated the association between language group (X: German=0 and Italian=1) and HL score (Y) (Figure 1).

Language group (X: German=0 and Italian=1) was the predictor, digital health information use the mediator (M), and the HL score the outcome (Y). Age, sex, educational attainment, self-rated health status, trust in professional health information, and trust in internet health information were entered as covariates in both equations. Indirect effects were estimated using 5000 bootstrap samples and percentile-based 95% CIs. A significant mediation effect was inferred when the CI for the indirect effect (*a*×*b*) did not include zero.

### Ethical Considerations

Ethical approval for this study was granted by the Institutional Review Board of the Institute of General Practice and Public Health, Bolzano, Italy (Protocol: 20/11/2023, November 20, 2023). The survey was incorporated into the 2024 Statistics Program of South Tyrol, which received approval from the Government of the Autonomous Province of Bolzano, Italy. The data collected for this research are protected under the Italian Statistics Act (Article 9, Legislative Decree 322/1989) and adhere to the regulations for personal data protection (EU Regulation 679/2016 and Legislative Decree 196/2003, as amended by Legislative Decree 101 of August 10, 2018). This study was conducted in accordance with the principles of the Declaration of Helsinki. Sampling and fieldwork were conducted by ASTAT within the official provincial statistics program; the research team received only deidentified records, and no information permitting identification of individual respondents was accessible during analysis. No financial compensation was provided.

Participation in this study was voluntary. Prior to completing the online questionnaire, participants provided informed consent. Completing the paper questionnaire and returning it by post was also regarded as an indication of informed consent. Although the survey adhered to official statistical procedures delineated by Italian law (Legislative Decree 322/1989), participants were not explicitly given the option to withdraw consent after completing the survey. Nevertheless, individuals retained the right to decline participation or request removal from the sample prior to submission.

## Results

### Sample Characteristics

**Figure 2.**
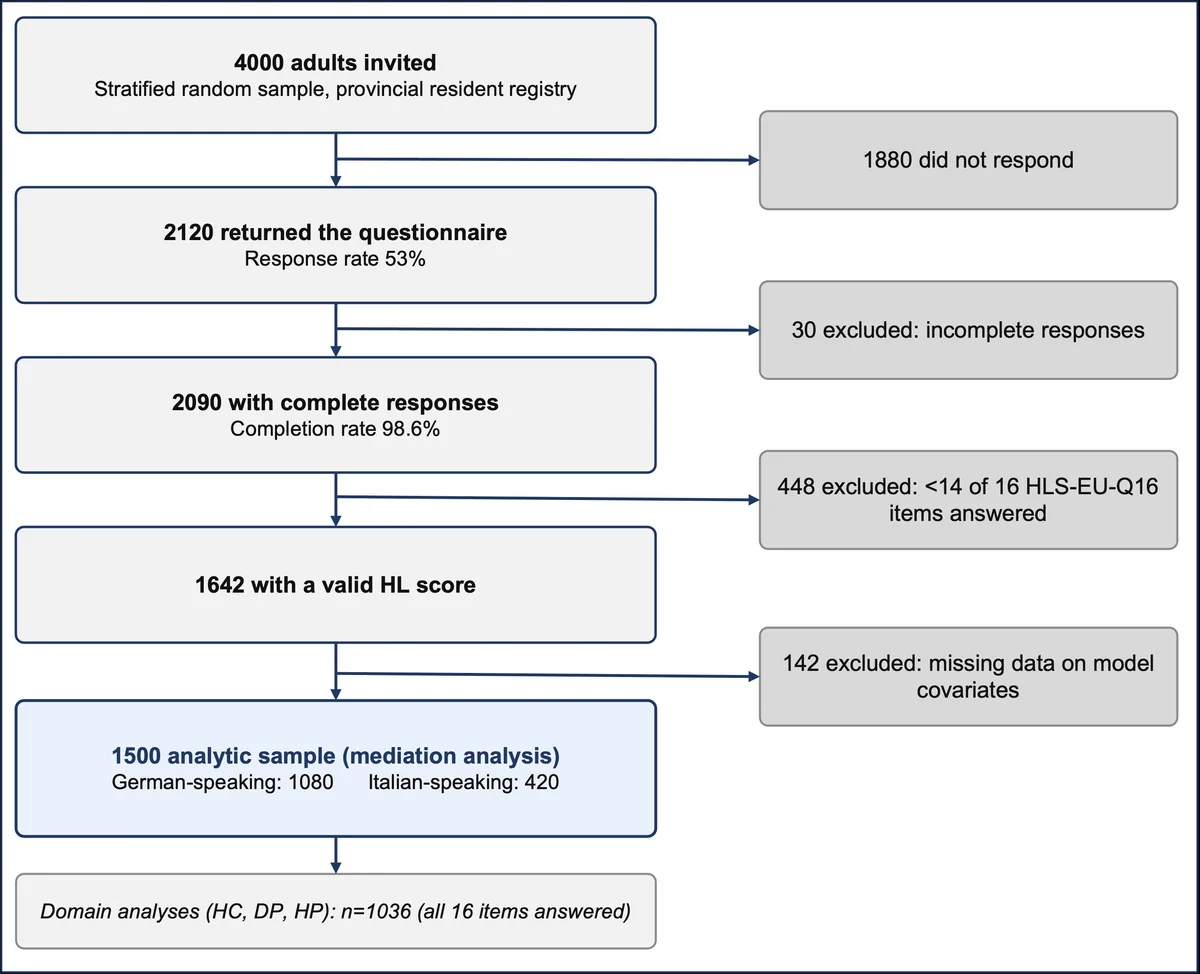
Flow of participants through the study from invitation to the analytic sample (mediation analysis) and to the domain subsample. DP: disease prevention; HC: health care; HL: health literacy; HLS-EU-Q16: 16-item Health Literacy Survey European Union Questionnaire; HP: health promotion.

Of the 4000 individuals invited, 2120 returned the questionnaire, yielding a response rate of 53% (2120/4000). Responses were collected anonymously. Among them, 2090 adults provided complete responses and were included in the analysis, corresponding to a completion rate of 98.6% (2090/2120). Cases with insufficient completion of the HLS-EU-Q16 instrument (ie, fewer than 14 of the 16 items answered) were excluded according to the standard scoring guidelines. After exclusion of cases with insufficient HLS-EU-Q16 completion, 1642 respondents remained. Of these, 142 had missing data on one or more model covariates and were excluded listwise, yielding a final analytic sample of 1500 participants (1080 German-speaking and 420 Italian-speaking) for the mediation analysis (Figure 2).

A post hoc power analysis was conducted using G*Power 3.1.9.6 (Heinrich-Heine-Universität) to estimate the statistical power for detecting a small effect with 7 predictors. Assuming a small effect size (*f*²=0.02), α=.05, and a total sample size of 1500, the analysis yielded a power exceeding 99%, indicating that the sample was well powered to detect the small effects examined in the mediation model.

The sample characteristics are presented in Table 1. Statistically significant differences were observed between German- and Italian-speaking participants across several sociodemographic and health-related variables.

**Table 1.** Sociodemographic and health characteristics of participants by language group^a^.

Characteristics	Total (N=1500)	German (n=1080)	Italian (n=420)	Test statistic (*df*)	*P* value	Effect size
Sex, n (%)				0.05 (1)^b^	.83	0.01
Male	679 (45.3)	487 (45.1)	192 (45.7)			
Female	821 (54.7)	593 (54.9)	228 (54.3)			
Age (y), mean (SD)	53.52 (17.27)	52.25 (17.32)	56.79 (16.71)	–4.68 (788.9)^c^	<.001	0.26
Age group (y), n (%)				18.15 (2)^b^	<.001	0.11
18‐34	268 (17.9)	213 (19.7)	55 (13.1)			
35‐54	459 (30.6)	346 (32.0)	113 (26.9)			
55‐99	773 (51.5)	521 (48.2)	252 (60.0)			
Residency, n (%)				386.08 (1)^b^	<.001	0.51
Urban	281 (18.7)	69 (6.4)	212 (50.5)			
Rural	1219 (81.3)	1011 (93.6)	208 (49.5)			
Education, n (%)				34.92 (3)^b^	<.001	0.15
Middle school or less	319 (21.3)	235 (21.8)	84 (20.0)			
Vocational school	483 (32.2)	390 (36.1)	93 (22.1)			
High school	384 (25.6)	252 (23.3)	132 (31.4)			
University or more	314 (20.9)	203 (18.8)	111 (26.4)			
Health or social sector worker, n (%)	176 (11.7)	127 (11.8)	49 (11.7)	0.00 (1)^b^	.96	0.00
Self-rated health (0-100), mean (SD)	76.86 (17.83)	77.89 (17.60)	74.20 (18.17)	3.57 (742.3)^c^	<.001	–0.21
Any chronic disease, n (%)	583 (38.9)	394 (36.5)	189 (45.0)	9.24 (1)^b^	.002	0.08
Number of chronic diseases (0-9), mean (SD)	0.58 (0.88)	0.53 (0.83)	0.70 (0.99)	–3.19 (657.5)^c^	.001	0.20
Trust in professional health information (4-16), mean (SD)	12.82 (2.00)	12.78 (2.04)	12.94 (1.90)	–1.47 (816.8)^c^	.14	0.08
Trust in internet health information (1-4), mean (SD)	2.09 (0.73)	2.10 (0.73)	2.06 (0.73)	0.86 (769.9)^c^	.39	–0.05
Digital health information use score (4-16), mean (SD)	8.28 (2.91)	8.14 (2.90)	8.64 (2.92)	–2.95 (760.4)^c^	.003	0.17
Health literacy score (0-16), mean (SD)	11.83 (3.46)	11.56 (3.48)	12.54 (3.31)	–5.10 (799.1)^c^	<.001	0.29
Health literacy domains (HLS-EU-Q16^d^ dichotomized sums), mean (SD)^e^						
Health care (0‐5)	3.72 (1.38)	3.59 (1.43)	4.04 (1.20)	–5.17 (637.7)^c^	<.001	0.33
Disease prevention (0‐4)	3.33 (0.95)	3.24 (1.00)	3.55 (0.79)	–5.26 (676.7)^c^	<.001	0.33
Health promotion (0‐7)	5.30 (1.70)	5.19 (1.71)	5.57 (1.63)	–3.34 (566.4)^c^	<.001	0.23

a Estimates are unweighted and describe the analytic sample of 1080 German-speaking and 420 Italian-speaking respondents (N=1500). Test statistic: Pearson *χ*2 for categorical variables and Welch *t* test (unequal variances) for continuous variables, with *df* in parentheses; Welch *df* are noninteger and reported to 1 decimal place. *P* values are from the same tests. Effect size: Cramér *V* (categorical) and Cohen *d* (continuous). Positive Cohen *d* indicates higher values among Italian speakers.

b *χ*² statistic.

c *t* test statistic.

d HLS-EU-Q16: 16-item Health Literacy Survey European Union Questionnaire.

e Health literacy domain scores are dichotomized item sums (higher=better): health care=items 2, 3, 4, 5, and 8; disease prevention=items 1, 6, 7, and 10; health promotion=items 9, 11, 12, 13, 14, 15, and 16. Per the manuscript protocol, domains were computed only for respondents answering all 16 items (n=1036).

The educational attainment, age distribution, and residency status differed significantly between the groups. Italian speakers were more frequently urban residents, older, and more likely to have completed a university education. German speakers had higher rates of vocational school attendance than Italian speakers. Italian-speaking participants reported lower self-rated health, a higher prevalence of chronic diseases, and a greater number of comorbidities. While trust in health information from health professionals and on the internet was comparable between groups, digital use scores were higher among Italian speakers. HL scores, both overall and in the domains of HC, DP, and HP, were also higher among Italian speakers. These differences were statistically significant, although the effect sizes were small. No significant group differences were found in sex distribution or employment in the health or social sectors.

### Correlates of Language Group, Digital Health Information Use, and HL

Pearson correlations between sociodemographic, health, trust, and information-related variables are shown in Table 2. HL showed a small positive correlation with education, subjective health status, and trust in internet health information, as well as with trust in health information from health care professionals. It was also positively correlated with the language group (Italian speakers) while showing a small negative correlation with the number of chronic diseases.

**Table 2.** Pearson correlations among sociodemographic, health, trust in internet health information, digital use of health information, and health literacy variables (N=1500)^a^.

Variables	Age	Educational level	Health status (0‐100)	Trust in internet health information	HL^b^ score	Italian	Trust in health care professionals	Number of chronic diseases	Sex	Digital use of health information
Age										
*r*	1	−0.335	–0.298	–0.266	0.002	0.118	0.041	0.413	0.001	–0.381
*P* value	—^c^	<.001	<.001	<.001	.94	<.001	.11	<.001	.98	<.001
Educational level										
*r*		1	0.194	0.149	0.095	0.142	0.030	–0.172	0.089	0.218
*P* value		—	<.001	<.001	<.001	<.001	.25	<.001	<.001	<.001
Health status (0‐100)										
*r*			1	0.129	0.221	–0.093	0.072	–0.445	0.031	0.126
*P* value			—	<.001	<.001	<.001	.005	<.001	.23	<.001
Trust in internet health information										
*r*				1	0.170	–0.022	0.107	–0.168	0.015	0.562
*P* value				—	<.001	.39	<.001	<.001	.57	<.001
HL score										
*r*					1	0.128	0.264	–0.112	0.042	0.106
*P* value					—	<.001	<.001	<.001	.10	<.001
Italian										
*r*						1	0.037	0.089	–0.006	0.076
*P* value						—	.15	<.001	.83	.003
Trust in health care professionals										
*r*							1	0.018	0.045	0.018
*P* value							—	.49	.08	.49
Number of chronic diseases										
*r*								1	0.003	–0.142
*P* value								—	.91	<.001
Sex										
*r*									1	0.057
*P* value									—	.03
Digital use of health information										
*r*										1
*P* value										—

a Educational level is dichotomized (high school or higher vs lower). Effect size interpretation for Pearson *r*: |*r*|<0.10=negligible; 0.10≤|*r*|<0.30=small; 0.30≤|*r*|<0.50=moderate; |*r*|≥0.50=large.

b HL: health literacy.

c Not applicable.

Language group membership (Italian vs German) was positively correlated with HL and was weakly but significantly associated with higher digital health information use. Digital use of health information was strongly associated with trust in internet health information and was moderately associated with younger age and a lower number of chronic conditions.

Age was broadly associated with other predictors, showing moderate-to-strong correlations with education, health status, the number of chronic diseases, and digital use. However, age showed no significant correlation with HL. Sex was minimally correlated with digital use but was unrelated to HL.

These findings informed the selection of covariates for the mediation analysis. Trust in internet health information emerged as a central correlate of digital health use.

### Mediation Analysis, Model Fit, and Explanatory Power

To investigate whether the use of digital health information mediates the relationship between language group (Italian vs German) and HL, a mediation analysis was conducted using PROCESS Model 4. The final model included the language group (X) as the predictor, the use of digital health information (M) as the mediator, and the HL score (Y) as the outcome. Covariates entered in both equations were age, sex, educational level, self-rated health status, trust in professional health information, and trust in internet health information. The results are presented in Table 3.

**Table 3.** Mediation analysis (PROCESS Model 4) of the effect of language group on health literacy through digital health information use (N=1500)^a^.

Outcome and predictor	Path	*B* (95% CI)	SE	*P* value
Digital health information use (mediator)^b^				
Language group (Italian)	*a*	0.718 (0.453 to 0.982)	0.135	<.001
Trust in internet health information	—^c^	1.973 (1.808 to 2.138)	0.084	<.001
Age	—	–0.042 (–0.050 to –0.034)	0.004	<.001
Sex (female)	—	0.282 (0.049 to 0.515)	0.119	.02
Educational level	—	0.258 (0.004 to 0.511)	0.129	.046
Self-rated health status	—	–0.002 (–0.008 to 0.005)	0.004	.65
Trust in professional health information	—	–0.046 (–0.104 to 0.012)	0.030	.12
Health literacy score (outcome)^d^				
Language group (Italian)	*c′*	0.961 (0.586 to 1.335)	0.191	<.001
Digital health information use	*b*	0.025 (–0.046 to 0.096)	0.036	.49
Trust in professional health information	—	0.385 (0.303 to 0.467)	0.042	<.001
Trust in internet health information	—	0.608 (0.337 to 0.879)	0.138	<.001
Self-rated health status	—	0.043 (0.033 to 0.052)	0.005	<.001
Age	—	0.020 (0.009 to 0.031)	0.006	<.001
Sex (female)	—	0.138 (–0.189 to 0.465)	0.167	.41
Educational level	—	0.250 (–0.106 to 0.606)	0.181	.17
Effect decomposition				
Total effect of language group	*c*	0.978 (0.607 to 1.350)	0.189	<.001
Direct effect of language group	*c′*	0.961 (0.586 to 1.335)	0.191	<.001
Indirect effect via digital use	*a*×*b*	0.018 (–0.035 to 0.074)	0.027^e^	—^f^

a Predictor (X): language group (German=0 and Italian=1). Mediator (M): digital health information use. Outcome (Y): health literacy score (16-item Health Literacy Survey European Union Questionnaire, range 0-16). Covariates were entered in both equations. Percentile bootstrap CIs based on 5000 resamples.

b Model *R*²=0.39; *F*_7,1492_=137.8; *P*<.001.

c Not available.

d Model *R*²=0.15; *F*_8,1491_=33.4; *P*<.001.

e Bootstrap SE.

f Significance of the indirect effect is inferred from the bootstrap CI; the CI includes zero, indicating no significant mediation.

The total effect of language group on HL was significant (*B*=0.98, 95% bootstrap CI 0.61 to 1.35), with Italian speakers showing higher HL scores than German speakers. Path *a* (effect of language group on digital health information use) was positive and significant (*B*=0.72; *P*<.001), indicating greater digital health information use among Italian speakers. However, path *b* (effect of digital health information use on HL) was not significant in the fully adjusted model (*B*=0.02; *P*=.49). Consequently, the indirect effect was small and nonsignificant, with a percentile bootstrap CI that included zero (*B*=0.018, 95% CI –0.035 to 0.074). Digital health information use therefore did not mediate the relationship between language group and HL. The direct effect of language group remained substantial and significant (*c′*: *B*=0.96; *P*<.001), indicating that the language-based HL difference is not accounted for by digital health information use. Among the covariates, trust in professional health information (*B*=0.39; *P*<.001), trust in internet health information (*B*=0.61; *P*<.001), self-rated health status (*B*=0.04; *P*<.001), and age (*B*=0.02; *P*<.001) were independent positive predictors of HL.

In summary, digital health information use did not mediate the association between language group and HL. Trust in professional and internet health information emerged as the more substantial correlates of HL, underscoring the role of informational trust over mere digital engagement.

The mediation model (Model 4) demonstrated an overall satisfactory explanatory power. The model predicting digital health information use (mediator M) accounted for 39% of the variance (*R*²=0.39; *P*<.001), indicating that the combination of language group, trust in internet health information, age, sex, educational level, self-rated health status, and trust in professional health information explains a substantial portion of the variance in digital information behavior.

For the outcome model predicting HL, the model explained 15% of the variance (*R*²=0.15; *P*<.001). Within this model, trust in professional health information, trust in internet health information, and self-rated health status were the strongest positive predictors, while digital health information use was not significantly associated with HL once these factors were accounted for.

## Discussion

### Principal Results

This study examined the association between language group membership and HL in a linguistically diverse region, focusing on whether the use of digital health information mediates this relationship. Consistent with previous research conducted in South Tyrol, Italian-speaking participants demonstrated higher HL scores than their German-speaking counterparts. Expanding on prior findings, the current analysis identified that the use of digital health information did not mediate language-related differences in HL: the indirect pathway was small and its CI included zero, whereas the direct language group effect remained robust. The comprehensive mediation model further revealed that lower trust in professional health information was independently associated with lower HL. These findings highlight the complex interplay between language group, digital engagement, and informational trust in shaping HL within a universal health care system.

### Comparison With Previous Literature

#### Language Group and HL

This study found that language group was significantly associated with HL in multilingual settings. Participants who spoke Italian exhibited higher HL levels than their German-speaking peers, as evidenced by superior overall scores and performance across the 3 evaluated domains: HC, DP, and HP.

Such disparities are not unique to South Tyrol. Speakers of nondominant languages more frequently report more effortful and less successful health-information seeking and tend to show lower competencies in the proactive domains of DP and HP while retaining relative strengths in health care navigation [20-25]. The higher scores of Italian speakers across all 3 domains in this study are consistent with this body of evidence.

While previous studies have raised concerns about the accuracy of HL assessments in multilingual populations due to translation challenges, differing health system experiences, and language structure (eg, phonetic vs logographic scripts) [26,27], these limitations do not apply to this study. Both Italian- and German-speaking participants completed linguistically validated versions of the HLS-EU-Q16 instrument, ensuring cultural and semantic equivalence across language groups.

Collectively, the current findings contribute to an expanding body of evidence indicating that language groups serve as significant and frequently underestimated sociocultural correlates of HL. The identified domain-specific differences highlight the necessity of culturally and linguistically tailored public health strategies that extend beyond educational attainment and socioeconomic status, addressing structural and perceptual barriers within multilingual communities.

#### Digital Health Information Use and Trust

In this study, participants who spoke Italian demonstrated significantly higher engagement with digital health information than their German-speaking counterparts, confirming earlier analyses based on the same dataset [13]. As expected, digital usage was moderately associated with younger age and higher educational attainment and weakly yet significantly correlated with overall HL. These associations are consistent with prior meta-analytic and population-level research, which indicates that digital engagement is influenced by sociodemographic factors, particularly age and education, and that it acts as a medium through which these factors affect HL [7,8].

Contrary to our initial hypothesis, the mediation model (Model 4) revealed that digital health information use did not serve as an important mediating pathway between the language group and HL. Although the model itself was statistically robust and the direct effect of the language group on HL remained substantial, the indirect effect through digital health information use was not statistically significant (the 95% bootstrapped CI included zero). This indicates that Italian speakers’ more frequent digital engagement does not sufficiently explain their higher HL levels relative to those of German speakers. This finding aligns with insights from the literature: although digital access and usage are necessary for the development of HL, they are not sufficient in isolation. The quality of digital engagement, individuals’ trust in online sources, and their capacity to critically evaluate and apply information are essential for converting digital behavior into significant improvements in HL [28,29]. Consequently, digital use may only partially mediate HL disparities under specific conditions, such as among individuals with high digital self-efficacy, advanced information-processing skills, or supportive social contexts that mitigate structural barriers [7,30].

Sbaffi and Rowley [16] and Mackert et al [31] demonstrated that individuals who exhibit higher levels of trust in online health sources are more inclined to convert digital health information into informed decisions and behaviors, particularly when these sources are perceived as credible and authoritative. Similarly, Seckin [32] found that trust in eHealth resources empowers older adults to act on health-related advice. In this study, trust in internet health information, entered as a covariate in the mediation model, was a strong predictor of both digital health information use and HL, with an association with HL that was stronger than that of digital use itself. However, it was unrelated to language group and therefore could not account for the language-based HL disparity. This may reflect the distinctive information environment of South Tyrol, where public health communication and access to professional sources are robust across language groups, potentially reducing reliance on digital sources alone.

As trust in internet health information was initially posited as a potential enhancer of the benefits associated with digital engagement, our fully adjusted models revealed a positive association with HL. This finding corresponds to previous research, which suggests that trust in digital sources facilitates the conversion of online information into effective health behavior [27-29]. Our findings indicate that digital health information use was not independently associated with HL once informational trust was accounted for and did not mediate the relationship between language group and HL. Moreover, trust in internet health information, while strongly predictive of digital use, was positively associated with HL and was not linked to the language group. This may suggest that in the South Tyrolean context, where public health communication and access to professional sources are robust, digital health information is used pragmatically and supplementarily rather than being the primary determinant of language-dependent literacy outcomes. Consequently, trust in online sources does not appear to differentiate individuals’ capacity to benefit from digital health engagement in terms of language-associated HL.

#### Other Predictors of HL

This study corroborates the finding that several well-established factors are associated with HL. Consistent with previous research, greater trust in health professionals emerged as a strong positive predictor of HL, highlighting the critical role of trust in institutional sources for effective engagement with health information [17,18]. Consistent with prior analyses of the same dataset, membership in the Italian-language group persisted as an independent predictor of higher HL, even when controlling for digital use and other covariates, indicating enduring language-based disparities in health information competency [12].

Digital health information use was weakly but positively associated with HL in bivariate analyses (*r*=0.11). In the fully adjusted model, however, this association was no longer significant once trust in internet health information and trust in professional sources were accounted for, suggesting that the bivariate association largely reflects shared variance with informational trust rather than an independent contribution of digital use frequency.

Previous research suggests that trust in digital sources can enhance the benefits of health information, but primarily when individuals possess the skills to evaluate source credibility and navigate complex online environments effectively [16,33]. Our findings similarly indicate that higher trust in internet health information is associated with better HL. This positive association may reflect the fact that individuals with higher HL are more capable of navigating and critically appraising digital health information, thereby fostering informed trust in online sources. In contexts with pervasive misinformation or a lack of guidance in assessing information quality, cultivating both critical appraisal skills and informed trust may be key to strengthening HL and mitigating informational vulnerabilities.

#### Implications for Public Health

The findings highlight that enhancing HL requires more than merely expanding digital access or increasing the quantity of available information. Public health interventions should prioritize enhancing individuals’ capacity to critically evaluate and apply health information while simultaneously fostering trust in both professional sources and reliable digital sources. This is particularly crucial in contexts where misinformation or digital overexposure may compromise health-related decision-making [34,35]. The current findings suggest that interventions should not only strengthen critical appraisal skills but also cultivate informed trust in credible online health information, as this trust is positively associated with higher HL.

Research on interventions underscores that culturally tailored, interactive, and theory-informed strategies are most effective in enhancing HL. Programs incorporating group discussions, bilingual materials, and skills-based learning components have shown particular benefits for socioeconomically disadvantaged and multilingual populations [36,37]. Moreover, interventions that actively involve patients and community members in both the design and delivery phases tend to be more relevant and sustainable, though such participatory approaches remain neglected in practice [38].

In South Tyrol, where linguistic group disparities persist despite a unified health care system, targeted HL interventions should address both structural barriers, such as linguistic accessibility, digital competence, and equitable access to digital resources, and perceptual factors, including information trust and evaluative confidence. Tailored regional strategies that leverage local language dynamics, patterns of digital use, and relationships of trust with professional and online sources are essential for promoting equitable health communication and improving HL outcomes in this multilingual setting [39,40].

### Limitations, Strengths, and Future Research

Several limitations constrain the interpretation of the findings of this study. First, the cross-sectional design precludes any causal inference regarding the relationships between language group, digital health information use, trust, and HL. Although mediation models can suggest pathways of association, the temporal ordering of these factors remains unclear [41]. Reverse pathways are equally plausible: individuals with higher HL may be more selective in their digital engagement and more discerning in whom they trust, thus the associations modeled here as predictors of HL may partly reflect its consequences. Second, although validated Italian- and German-language versions of the HLS-EU-Q16 were used, unmeasured cultural factors may have influenced both HL and digital engagement. Despite linguistic validation, the conceptual equivalence of HL assessments across cultural and social contexts cannot be fully assured [42].

Furthermore, the measurement of digital usage captured frequency but did not account for the depth, quality, or the critical evaluation of information. Similarly, trust was assessed using single items or brief indices, which may oversimplify a multidimensional construct that evolves through experience and context. Additionally, approximately 21% (448/2090) of respondents had incomplete responses to the HLS-EU-Q16 and were excluded, potentially introducing selection bias and affecting the generalizability of the HL estimates [13].

Although age was statistically controlled for in all multivariate models, the higher average age of the Italian-speaking cohort may have introduced residual confounding. Age is intricately associated with digital engagement and trust dynamics, potentially attenuating or obscuring the stronger associations between language group, digital use, and HL.

The sample was limited to the 2 predominant language groups in South Tyrol, thereby excluding Ladin speakers, migrants, and other minority populations in South Tyrol. This restriction constrains the generalizability of the findings to broader multilingual and multicultural contexts.

Despite these limitations, this study has several strengths. It is based on a large, population-based sample with a high response rate, enhancing the generalizability of findings within the South Tyrolean context. The use of validated HL instruments in both German and Italian ensures linguistic comparability. Additionally, the comprehensive analysis, including mediation modeling and consideration of trust and digital engagement, provides novel insights into the complex interplay between language, digital behaviors, and HL in a multilingual region.

Future research should encompass a broader range of linguistic and cultural groups and integrate variables such as migration background, acculturation, and digital competencies. Longitudinal or mixed methods studies could elucidate the temporal dynamics between digital engagement, trust, and HL development. Using more nuanced measurements of trust, including multidimensional scales, alongside assessments of digital information quality and processing skills would yield a deeper understanding of these relationships. Addressing item nonresponse through multiple imputation and examining patterns of missingness may also enhance the robustness and representativeness of HL assessment.

### Conclusions

This population-based study from South Tyrol confirms that language group membership is a critical social determinant of HL in multilingual regions. Italian-speaking participants exhibited higher HL levels and more frequent engagement with digital health information than their German-speaking counterparts. However, the use of digital health information did not significantly mediate the observed differences in HL between the language groups. Moreover, trust in internet health information, while strongly predictive of digital use, was positively associated with HL but did not contribute to explaining the language-based disparities.

These findings suggest that while digital engagement and trust in online sources are positively linked to HL, they are insufficient on their own to overcome the structural and cultural barriers that contribute to language-based disparities. A more comprehensive strategy that integrates digital engagement with strengthened critical appraisal skills and targeted support for minority language groups is necessary to address these inequalities.

Therefore, addressing HL inequalities requires public health strategies that go beyond enhancing digital access. Interventions must focus on strengthening critical appraisal skills, promoting digital HL, and fostering trust in professional health information sources. In multilingual regions such as South Tyrol, tailored approaches that account for linguistic, cultural, and informational contexts are essential for equitable health care communication.

Future research should use longitudinal and mixed methods designs to better understand the temporal dynamics among digital engagement, trust, and the development of HL. Including underrepresented linguistic minorities and incorporating multidimensional measures of trust, digital literacy, and cultural orientation will be vital to informing effective, equity-oriented health policies and education in diverse societies.

## Abbreviations

ASTAT: Provincial Institute of Statistics of the Autonomous Province of Bolzano-South Tyrol
DP: disease prevention
HC: health care
HL: health literacy
HLS-EU-Q16: 16-item Health Literacy Survey European Union Questionnaire
HP: health promotion
